# Uterus proliferative period ceRNA network of Yunshang black goat reveals candidate genes on different kidding number trait

**DOI:** 10.3389/fendo.2023.1165409

**Published:** 2023-05-12

**Authors:** Xiaolong Du, Yufang Liu, Xiaoyun He, Lin Tao, Meiying Fang, Mingxing Chu

**Affiliations:** ^1^ Key Laboratory of Animal Genetics, Breeding and Reproduction of Ministry of Agriculture and Rural Affairs, Institute of Animal Science, Chinese Academy of Agricultural Sciences, Beijing, China; ^2^ Department of Animal Genetics and Breeding, National Engineering Laboratory for Animal Breeding, Ministry of Agriculture and Rural Affairs (MARA) PRC Laboratory of Animal Genetics and Breeding, College of Animal Science and Technology, China Agricultural University, Beijing, China

**Keywords:** goat, uterus, proliferative period, ceRNA network, cell adhesion

## Abstract

Pregnancy loss that occurs in the uterus is an important and widespread problem in humans and farm animals and is also a key factor affecting the fecundity of livestock. Understanding the differences in the fecundity of goats may be helpful in guiding the breeding of goats with high fecundity. In this study, we performed RNA sequencing and bioinformatics analysis to study the uterus of Yunshang black goats with high and low fecundity in the proliferative period. We identified mRNAs, long non-coding RNAs (lncRNAs), and microRNAs (miRNAs) by analyzing the uterine transcriptomes. The target genes of the identified miRNAs and lncRNAs were predicted, and miRNA–mRNA interaction and competitive endogenous RNA (ceRNA) networks were constructed. By comparisons between low- and high-fecundity groups, we identified 1,674 differentially expressed mRNAs (914 were upregulated, and 760 were downregulated), 288 differentially expressed lncRNAs (149 were upregulated, and 139 were downregulated), and 17 differentially expressed miRNAs (4 were upregulated, and 13 were downregulated). In addition, 49 miRNA–mRNA pairs and 45 miRNA–lncRNA pairs were predicted in the interaction networks. We successfully constructed a ceRNA interaction network with 108 edges that contained 19 miRNAs, 11 mRNAs, and 73 lncRNAs. Five candidate genes (PLEKHA7, FAT2, FN1, SYK, and ITPR2) that were annotated as cell adhesion or calcium membrane channel protein were identified. Our results provide the overall expression profiles of mRNAs, lncRNAs, and miRNAs in the goat uterus during the proliferative period and are a valuable reference for studies into the mechanisms associated with the high fecundity, which may be helpful to guide goat to reduce pregnancy loss.

## Introduction

In female agricultural animals, fecundity traits are the main indicator of their economic value, especially the number of fetuses (1). Pregnancy loss that occurs in the uterus is an important and widespread problem in humans and farm animals, especially in low-ovulatory animals (2). During natural conception, approximately 75% of the final pregnancy failures are due to the inappropriate environment in the uterus (3, 4). Differences between uteruses are the main consideration when exploring pregnancy loss. The Yunshang black goat is a specialist mutton goat that was bred in China. These goats have good economic benefits, but intravarietal kidding numbers vary significantly (5). Hence, it is important to study the effect of uterine variability on kidding traits.

The uterus comprises the serous membrane, myometrium, and endometrium. The endometrium consists of luminal and glandular epithelial cells surrounded by supporting stromal cells (6). The main function of the uterus is to maintain pregnancy, and the endometrium promotes implantation and the decidua and supports the growth and development of the embryo (7). Embryo implantation in humans and rodents mainly involves embryonic trophectoderm cells penetrating the endometrial epithelial layer and invading the stroma to form the placenta and establish pregnancy (8). However, the pregnancy process in ruminants is entirely different from that of humans and rodents. In ruminants, trophoblast cell fusion involves the development of finger-like villi or papillae between the caruncles of the uterus. These structures penetrate the mouths of superficial ducts of the uterine glands at days 15–18 after mating. Although similar features were described for the cow conceptus from day 15 of pregnancy, goats did not exhibit the same phenomenon (9, 10). The main difference is that, in goats, superficial implantation occurs; that is, the embryonic trophectoderm cells adhere to the endometrial caruncle and endometrial epithelial cells fuse to form multinucleated syncytial plaques, then connect with each other to form a cotyledon type placenta (11). Complex mucins bind the embryo to the uterus. A study using the uterine gland knockout (UGKO) ewe model found that the reduction or absence of endometrial-derived cohesin was the main cause of recurrent miscarriage in UGKO ewes (12). The endometrium proliferates and secretes with the cyclical changes in the ovary, and whether embryo implantation is successful is directly related to regression of the corpus luteum and recovery of the endometrium (13). However, some scientists consider the uterus to be the cause of the cyclical changes in the ovary because the uterus can secrete prostaglandins to promote luteolysis. Indeed, when the uterus was removed during the luteal phase, the estrus period was delayed by 5 months, which is the same as the pregnancy time of goats (10).

MicroRNAs (miRNAs) that were differentially expressed in the proliferative and secretory phases of human endometrial were reported in 2008 (14). Subsequent research found that changes in miRNA expression levels in endometrial tissue may be influenced by estradiol and progesterone, indicating that miRNAs may be involved in the reproductive physiological processes of the endometrial tissue (15). The involvement of miR-182 in the establishment of endometrial receptivity by regulating the pleiotrophin (*PTN*) and homeobox A10 (*HOXA10*) genes was found in dairy goat endometrial epithelial cells (16, 17). More recently, the roles of long non-coding RNAs (lncRNA) in diverse biological activities, such as embryogenesis and transcriptional regulation, have been recognized, and many lncRNAs with functions in goat endometrium during pregnancy recognition have been discovered (18, 19). However, that lncRNAs and miRNAs jointly affect mRNA expression in goat endometrium has rarely been reported. In this study, we performed miRNA and lncRNA sequencing and constructed an miRNA–lncRNA–mRNA interaction network and competitive endogenous RNA (ceRNA) network to explore the factors that affect goat kidding numbers from a new perspective.

## Materials and methods

### Ethics statement

Ethics approval (no. IAS2019-63) was granted by the Animal Ethics Committee of the Institute of Animal Sciences of Chinese Academy of Agricultural Sciences (IAS-CAAS) (Beijing, China).

### Sample collection, RNA extraction, and quality analysis

A total of 10 non-pregnant adult female Yunshang black goats (aged 3–5 years old; weight, 41–63 kg) were selected randomly from the Yixingheng Animal Husbandry Technology Co., Ltd, Tuanjie Township Base in Kunming City (24° 23′ north latitude), Yunnan Province, China, and were used in this study. The goats were separated into a high-fecundity group (HF group; average kidding number, 3.00 ± 0.38, n=5) and a low-fecundity group (LF group; kidding number, 1.32 ± 0.19, n=5) (p <0.01, t-test) according to their kidding number records. All uterine tissue layers from similar segments with distance from the cervix were collected in the proliferative period after synchronous estrus processing and stored at −80°C until used for RNA extraction. All pretreatments were based on those of a previous study (20). Blood samples were collected for hormone determination, which was measured by competitive radioimmunoassay.

Total RNA was isolated from 10 uterine tissue samples (containing endometrium and stroma) with TRIzol (Invitrogen, Carlsbad, CA, USA) according to the manufacturer’s protocol. The quality of the RNA samples was determined using a NanoDrop 2000 spectrophotometer (Thermo Scientific, Wilmington, DE, USA), Agilent Bioanalyzer 2100 system (Agilent Technologies, Palo Alto, CA, USA), and RNA Nano 6000 Assay (Agilent Technologies, CA, USA). The OD 260/280 was 1.8–2.2, and the RNA integrity number (RIN) of all samples was >7.

### lncRNA, mRNA, and miRNA library preparation and sequencing

An Epicenter Ribo-Zero™ Removal Kit (Epicenter, Madison, WI, USA) was used to remove rRNA, and rRNA-free residues were cleaned by ethanol precipitation. Then, sequences that passed the quality inspection were used for library construction and sequencing. The lncRNA and mRNA library was constructed using 3 μg of the total RNA and a NEBNext^®^ UltraTM Directional RNA Library Prep Kit (NEB, Ipswich, MA, USA) according to the manufacturer’s instructions. The Illumina NovaSeq 6000 platform (Illumina, San Diego, CA, USA) was used for PE150 (paired-end 150 bp) sequencing. The miRNA library was built using a Small RNA Sample Prep Kit (Tiangen, Beijing, China). The total RNA was used as a template, and cDNA was synthesized with a special adapter. After PCR amplification, the products were purified by polyacrylamide gel electrophoresis (PAGE), and 140–160-bp sequences were recovered to construct an miRNA sequencing library. The above platforms were used for SE50 (single-end 50 bp, SE50) sequencing. All the sequencing were outsourced to Wuhan Frasergen Gene Information Co., Ltd. (Wuhan, China).

### Data processing and transcriptome assembly

The original image file from the sequencer was converted into a sequence file that contained mainly sequence information and sequencing quality information using CASAVA software. The raw data (FASTQ) were filtered for use in subsequent analysis as follows: 1) reads that contained the adapters were removed; 2) reads with N content >1% of the length of the read itself and reads that constituted the paired-end pair were filtered out; and 3) reads with low-quality bases (≤20) that exceeded 50% of the length of read itself and reads that constituted the paired-end pair were filtered out.

Quality evaluation of the clean reads included calculation of sequencing quality (Q20 and Q30) base distribution (GC content). Clean reads that passed the quality evaluation were mapped to the reference genome (*Capra hircus*, ARS1) using HISAT2 (v2.1.0). StringTie (v1.3.5) was used to assemble the transcripts (21, 22). HISAT2 was run with parameters “-x -no-unal -un-conc”, “-rna - strandness RF” and “-dta -t -p 4,” String Tie with “-e -B”, “-G ref.gtf -rf -1;” the other parameters were set as the default. The fragments per kilobase per million reads (FPKMs) for each gene were calculated based on the length of the gene and read counts mapped to the gene (23).

### Identification of mRNA, lncRNA, and miRNA

We screened the assembled transcripts using the following criteria to identify lncRNAs and mRNAs: 1) transcript lengths ≥200 bp and number of exons >1; 2) transcripts were compared with mRNA and other non-coding RNA sequences (rRNA, tRNA, snRNA, and snoRNA) in known databases using Cuffcompare, and rRNA and tRNA sequences were removed (23). When an annotated lncRNA overlapped the exon region of a sequence in the database, it was included as a known lncRNA in the subsequent analysis (3). For novel lncRNAs and mRNAs, three coding potential prediction software [CNCI (24), CPC2 (25), and PLEK (26)] were used to predict the coding potential of the transcripts, and the intersection of the three prediction results was used.

To identify miRNAs, clean high-quality reads of 18–35 nt were compared against miRNA sequences in the miRBase (v22) database (27) using Bowtie2. Clean high-quality reads not identified as known miRNA sequences were compared with other non-coding RNA sequences (rRNA, tRNA, snRNA, and snoRNA) in Rfam (14.2) (28) and classified and annotated. RepeatMasker was used to identify repeat sequences in the clean reads that were not annotated as non-coding RNAs or known miRNAs (29). After evaluating these sRNAs and using the location information of exons and introns of the gene from the matching (100% positional overlap) results, the sRNAs were annotated (30). For the sRNA reads that did not match the above-known annotation type, miREvo and miRDeep2 (31) were used to predict novel miRNAs, and the sequence, expression, and structure information of these miRNAs were analyzed. Sequences with different core sequences, precursor sequences, and positions were considered to be new miRNAs.

### Expression level analysis and identification of DE lncRNA, mRNA, and miRNA

The count of reads mapped to each transcript was obtained using StringTie and normalized by computing TPM and FPKM values to indicate miRNA and mRNA/lncRNA expression levels. For the paired-ended sequencing, paired-end reads from the same fragment were counted as one fragment. The DESeq2 R package was used for differential expression analysis, and adjusted p <0.05 and |log2FC| >1 were used as the threshold (32).

### GO and KEGG enrichment analyses

The Gene Ontology (GO) and Kyoto Encyclopedia of Genes and Genomes (KEGG) functional analyses of the target genes of the differentially expressed lncRNAs (DELs) and differentially expressed miRNAs (DEMs), and the differentially expressed mRNAs (DEGs) were performed using GOSeq (Release 2.12) and KOBAS (v2.0) (33), respectively. The hypergeometric test method was implemented to assess significantly enriched GO terms and KEGG pathways, and p-values (t-test) <0.05 were considered significant.

### Association analysis of lncRNA–miRNA–mRNA analysis

To analyze the interaction between miRNA and mRNA, we used MiRanda and qTar to predict the target genes of the miRNAs and used the intersection of the two software as the result (34). Correlated miRNA–mRNA pairs were identified by Pearson correlation analysis. Because lncRNAs can act as miRNA sponges, they can inhibit the regulatory effect of miRNAs on their target mRNAs; therefore, we analyzed the relationship between miRNAs and lncRNAs in a similar way.

### Verification of miRNA, lncRNA, and mRNA expression profiles with RT-qPCR

For the RT-qPCR analysis, we used a PrimeScript™ RT Reagent Kit (TaKaRa, Dalian, China) and miRcute Plus miRNA First-Strand cDNA Kit (TIANGEN) synthetic-detection templates. The expression of the selected mRNAs and miRNAs was quantitatively analyzed using a SYBR Green qPCR Mix Kit (TaKaRa) and miRcute Plus miRNA qPCR Kit (TIANGEN) according to the manufacturer’s instructions. The RT-qPCRs were performed on a RocheLight Cycler^®^480 II system (Roche Applied Science, Mannheim, Germany). The PCR primers were synthesized by Shanghai Sangon Biotech. The relative expression was calculated by the 2^−ΔΔCP^ method. The goat PRL19 gene and U6 were used as reference genes. The primer sequences are listed in **Supplementary Material**.

### Statistical analysis

The SPSS 20.0 software was used for statistical analysis. The statistical significance of the data was tested by performing paired *t*-tests. The results are presented as the means ± SEMs of three replicates, and statistical significance was represented by *p-*values (*p < 0.05; **p<0.01).

## Results

### Results of phenotypic determination and hormone level determination

Test samples were selected based on an evaluation of kidding records over two birthings. At the time of sample collection, we also recorded body weight data, hormone levels, and total number of follicles in the left and right ovaries after dissection. The phenotypic data for the five high-fecundity and five low-fecundity Yunshang black female goats in this study are shown in **Table 1**. Except for significant phenotypic date, kidding number, and average kidding number (p <0.01, t-test), some not significant phenotypic differences are listed in **Table 1** between the high- and low-fecundity groups (*p*>0.05, t-test).

**Table 1:** Summary of the Yunshang black she-goats phenotypic data.

Performance	Weight	Kidding N1/N2	Average	Number of follicles	E2 pg/ml	P ng/ml
LF1	62.70	2.00/1.00	1.50	36.00	285.62	0.01
LF2	56.00	2.00/2.00	2.00	2.00	410.33	0.04
LF3	51.50	1.00/2.00	1.50	32.00	293.19	0.05
LF4	50.20	2.00/2.00	2.00	38.00	251.26	0.04
LF5	62.00	1.00/2.00	1.50	42.00	234.65	0.02
HF1	54.00	3.00/3.00	3.00	43.00	271.17	0.03
HF2	59.00	3.00/3.00	3.00	43.00	551.17	0.03
HF3	48.30	3.00/4.00	3.50	31.00	317.48	0.05
HF4	56.70	3.00/3.00	3.00	63.00	324.07	0.01
HF5	41.70	3.00/3.00	3.50	28.00	249.37	0.05
T-test *p*	0.295	0.001/0.001	0.000	0.255	0.465	0.884

LF and HF, respectively, represent the low- and high-fecundity groups. Kidding N1 and Kidding N2 represent the number of kids born in the first and second births, respectively.

### Overview of sequencing quality control results in uterine tissues

Total RNA was extracted from 10 uterine tissues and used to construct two sets of libraries: one to identify lncRNAs and mRNAs, and the other to identify small RNAs (sRNAs). After processing and quality control of the raw data, an average of 118.3 million and 24.7 million clean reads were obtained from the lncRNA/mRNA and sRNA libraries, respectively (**Table 2**). The mapping rates of the lncRNA/mRNA and sRNA reads against the goat genome (*Capra hircus*, ARS1) were an average of 96.97% and 97.72%, respectively. The quality of the lncRNA and mRNA sequencing results was assessed by calculating the Q30 and GC content of the two reads that constituted the paired-ends. The results showed that the quality of the clean reads met the requirements for subsequent analysis. The other filtering criteria used to identify candidate lncRNAs, mRNAs, and miRNAs are provided in **Supplementary Material S1**.

**Table 2:** Summary of the mapping data from the uterine tissues.

	Clean reads		Mapped rate (%)		Q30 (%)		GC (%)	
	lncRNA/mRNA	sRNA	lncRNA/mRNA	sRNA	lncRNA/mRNA	sRNA	lncRNA/mRNA	sRNA
HF-1	114278598	24045828	97.22%	97.87%	95.9;93.8	97.75%	50.1;51.2	49.36%
HF-2	140788452	25692487	96.74%	97.95%	95.7;93.4	97.82%	49.6;50.6	49.49%
HF-3	114783120	27958571	96.86%	97.31%	95.8;94.2	97.84%	50.3;51.3	49.24%
HF-4	100002864	24034570	96.96%	97.69%	95.8;94.9	97.67%	49.8;50.7	49.51%
HF-5	146338162	25945228	97.10%	97.18%	95.2;95.1	97.84%	49.4;50.3	49.31%
LF-1	116625272	23683076	96.86%	97.31%	95.7;93.5	96.54%	50.9;51.9	49.30%
LF-2	126602416	25309418	97.29%	97.76%	95.2;95.5	97.46%	51.0;52.3	49.29%
LF-3	116408604	24220024	96.97%	98.08%	95.7;94.6	97.34%	50.0;51.1	49.59%
LF-4	105324250	24148646	96.56%	97.88%	95.1;95.0	97.42%	49.1;50.0	49.34%
LF-5	101908238	22093907	97.14%	98.23%	95.3;95.1	97.45%	50.1;51.3	49.72%

LF and HF represent the low-fecundity group and the high-fecundity group, respectively. The data of lncRNA/mRNA in the Q30 (%) and GC (%) lists show the result of the assessment of two reads that constitute the paired end, respectively.

### Identification of lncRNA, mRNA, and miRNA in uterine tissues

**Figure 1:**
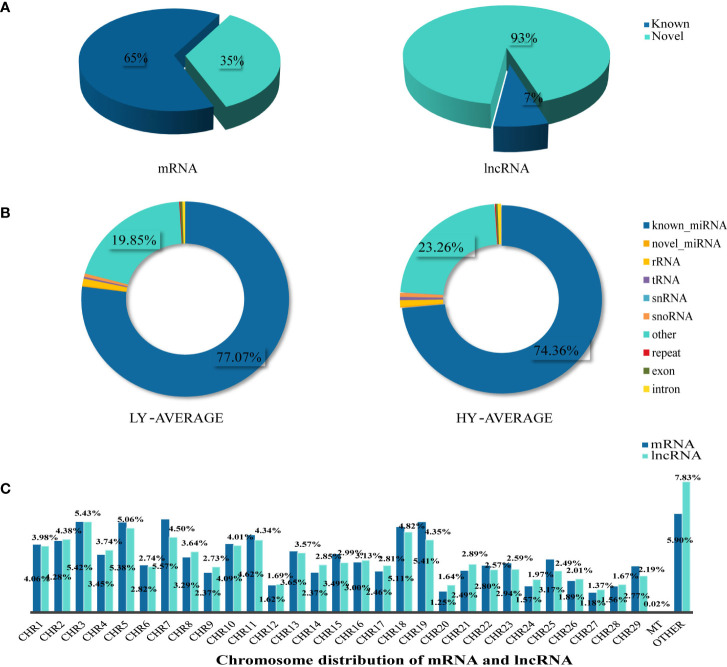
Summary information of lncRNA, mRNA, and miRNA identification. **(A)** According to the identification results, we mainly divided lncRNA and mRNA into Known and Novel two types, and the pie chart was made according to the proportion. **(B)** The statistical results of unique clean reads from the uterine tissues in the two experimental groups. We distinguished small RNAs into 10 types as shown in the figure. **(C)** Chromosome distribution of identified mRNA and lncRNA from the uterine tissues.

A total of 67,424 mRNA were identified and successfully mapped to the goat genome. StringTie was used to compare these mRNAs to the reference annotations in the goat genome. The results showed that 65% of the mRNAs were annotated in the reference database; therefore, we defined them as known RNAs. The remaining mRNAs (35%) for which no annotations were found were defined as novel mRNAs. We used the same method to identify sequences longer than 200 bp with more than two exons as lncRNAs. We found that only 7% of the identified lncRNAs were known lncRNAs; the remaining 93% were defined as novel lncRNAs (**Figure 1A**). The predicted genomic position of the novel lncRNAs in the goat genome showed that 8,648 were intergenic lncRNAs, 12,389 were intronic lncRNA, and 2,928 were antisense lncRNA (**Supplementary Material S2**). We used StringTie to assemble the annotated reads into transcripts so that their detailed chromosome locations could be obtained. We found that the proportions of mRNAs and lncRNAs were similar on most of the chromosomes. Among the autosomal chromosomes, chromosomes 3, 5, 7, 18, and 19 had the highest proportions of mRNAs and lncRNAs, followed by chromosomes 1, 2, 10, and 11. Chromosomes 12, 20, 24, 27, and 28 had the lowest proportions of mRNAs and lncRNAs. We also found that 0.02% of the transcripts were mitochondrial mRNAs (n=13); no mitochondrial lncRNAs were identified (**Figure 1C**).

To identify miRNAs, we compared the sequenced reads against a number of sRNA databases that had different annotation information and defined the sRNAs in the following order: known miRNAs > rRNAs > tRNAs > snRNAs > snoRNAs > repeats > novel miRNAs > exons > introns. We found that known miRNA accounted for 74.36% and 77.07% of the average total sRNAs in the high- and low-fecundity groups, respectively. Other sRNAs (tRNA, rRNA, snRNA, and snoRNA), which were identified by comparisons against known databases such as Rfam, accounted for a very small proportion of the average total sRNAs in the two groups. Most of the remaining sRNAs accounted for 23.26% and 19.85% of the average total sRNAs in the high- and low-fecundity groups, not including repeats or degraded fragments of genomic exons and introns, which accounted for very small proportions of the average total sRNAs in the two groups (**Figure 1B**; **Supplementary Material S2**).

### Results of expression level analysis of mRNA, lncRNA, and miRNA

**Figure 2:**
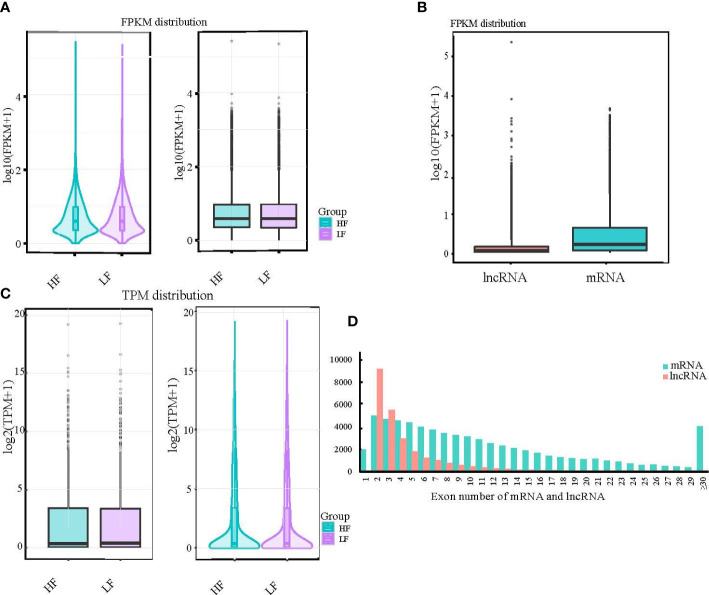
Results of expression level analysis of mRNA, lncRNA, and miRNA. **(A, B)** The expression levels of different experimental groups were compared by violin and box diagrams of all transcripts of FPKM and TPM. For the same group of repeated samples, the final FPKM and TPM values were the average of all duplicated data. **(C)** Box diagrams were also created to show the expression levels of different types of transcripts. **(D)** Additionally, a comparative histogram was generated to show the number of exons in different types of transcripts.

To normalize the counts for the clean paired-end reads, we calculated FPKM and transcripts per kilobase million (TPM) to obtain the expression levels of the transcripts for subsequent quantitative analysis. We found that the overall distribution of transcripts in the high- and low-fecundity groups was almost the same, whether by maximum, minimum, or median analysis (**Figures 2A, B** **)**. The consistency of the expression data also confirmed the accuracy of our sequencing data. The FPKM distribution of the lncRNAs and mRNAs was significantly different but in line with the expected result (**Figure 2C**). The number of exons in the lncRNA and mRNA transcripts was very different; most of the lncRNAs had two exons, whereas most of the mRNAs had from two to seven exons (**Figure 2D**; **Supplementary Material S3**).

### Differential expression analysis of mRNAs, lncRNAs, and miRNAs

**Figure 3:**
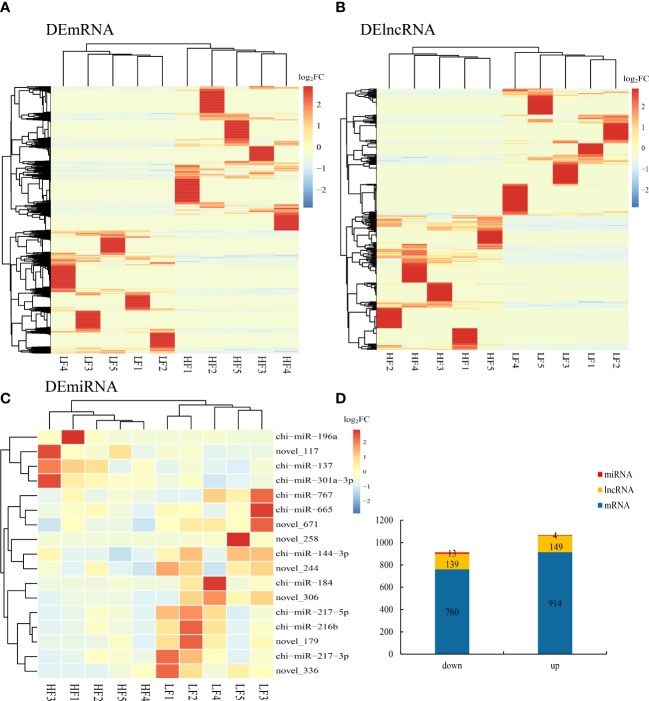
Differentially expressed genes (DEGs), lncRNA (DELs), and miRNAs (DEMs) in goat uterine. **(A)** Comparison of differentially expressed genes (DEGs) represented as a heatmap. **(B)** Comparison of differentially expressed lncRNA (DELs) represented as a heatmap. **(C)** Comparison of differentially expressed miRNAs (DEMs) represented as a heatmap. **(D)** Stacked column chart showing the distribution of DEGs, DELs, and DEMs in the comparison.

We identified differentially expressed lncRNAs (DELs) and mRNAs (DEGs) by comparing the average expression levels between the low- and high-fecundity groups with a cutoff threshold of |log2fold change (FC)| ≥1 and q <0.05. A total of 1,674 DEGs and 288 DELs were identified; 914 DEGs were upregulated and 760 DEGs downregulated, and 149 DELs were upregulated and 139 DELs downregulated. We also identified 17 DEMs (four were upregulated and three were downregulated) in the low-fecundity vs. high-fecundity comparison with a cutoff threshold of |log2FC| ≥1 and p <0.05 (**Figure 3D**). The Euclidean distance was calculated based on log base 2 of the DEG, DEL, and DEM expression levels in each sample, and then, the overall clustering results of the samples were obtained using a systematic hierarchical clustering method (**Figures 3A–C**; **Supplementary Material S4**).

### GO and KEGG enrichment analysis

**Figure 4:**
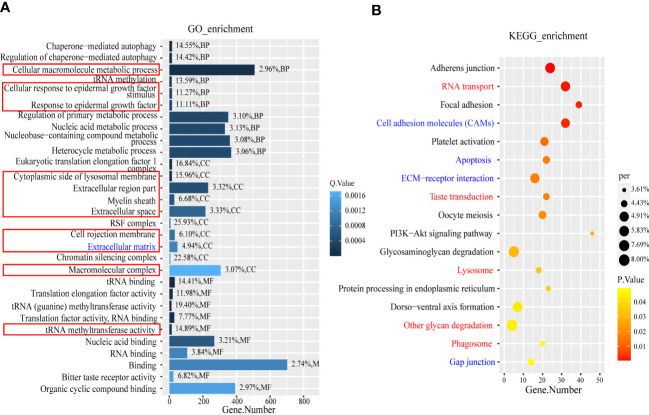
The GO term and KEGG enrichment analysis of DEGs and target genes of DEMs and DELs. **(A)** The part in the red box is the BP term (GO: cellular macromolecule metabolic process, GO: cellular response to epidermal growth factor stimulus, and GO: response to epidermal growth factor), CC term (GO: cytoplasmic side of lysosomal membrane, GO: extracellular region part, GO: myelin sheath, GO: extracellular space, GO: cell projection membrane, GO: extracellular matrix, and GO: macromolecular complex) and MF term (GO: translation factor activity, RNA binding). The blue font is CC term (GO:0031012). **(B)** The red font represents the overlapping pathway enriched by DEGs and DELs target genes, while the blue font represents the overlapping pathway enriched by DEGs and DEMs target genes.

GO and KEGG enrichment analysis were performed using the DEGs and the target genes of the DELs and DEMs. The enrichment results were screened using different thresholds (GO, q ≤0.01; KEGG, p ≤0.05) because the numbers of enriched GO and KEGG terms were very different (DEGs GO, n=124; KEGG, n=17; target genes of DELs GO, n=1,135; KEGG, n=100; target genes of DEMs GO, n=304; KEGG, n=41.). By overlapping the enriched GO terms for the DEG and DEL target genes, we identified 54 terms that were common to both, whereas only four GO terms for the DEG and DEM targets overlapped. The top 10 overlapping terms under the three main GO categories, namely, biological process, cellular component, and molecular function, were analyzed, and the results are shown in the column chart in **Figure 4A**. They include two biological process terms (GO: cellular macromolecule metabolic process, GO: cellular response to epidermal growth factor stimulus), eight cellular component terms (GO: response to epidermal growth factor, GO: cytoplasmic side of lysosomal membrane, GO: extracellular region part, GO: myelin sheath, GO: extracellular space, GO: cell projection membrane, GO: extracellular matrix, GO: macromolecular complex), and one molecular function MF term (GO: translation factor activity, RNA binding). It is worth noting that the cellular component term extracellular matrix and the biological process term cellular response to endogenous stimulus, which are not listed in **Figure 4A**, overlapped with terms in the top 10 overlapping terms under the three main GO categories. We constructed a bubble chart based on all the enriched KEGG pathways and found that only 17 pathways were significantly enriched (p ≤0.05) (**Figure 4B**); nine and eight of these pathways were for the DEL and DEM target genes, respectively. Notably, four of these pathways were significantly enriched for the DEGs and DEL and DEM target genes, namely, adherens junction, focal adhesion, platelet activation, and PI3K-Akt signaling pathway (**Supplementary Materials S5**, **S6**).

### Interaction regulation network about miRNA–lncRNA–mRNA

**Figure 5:**
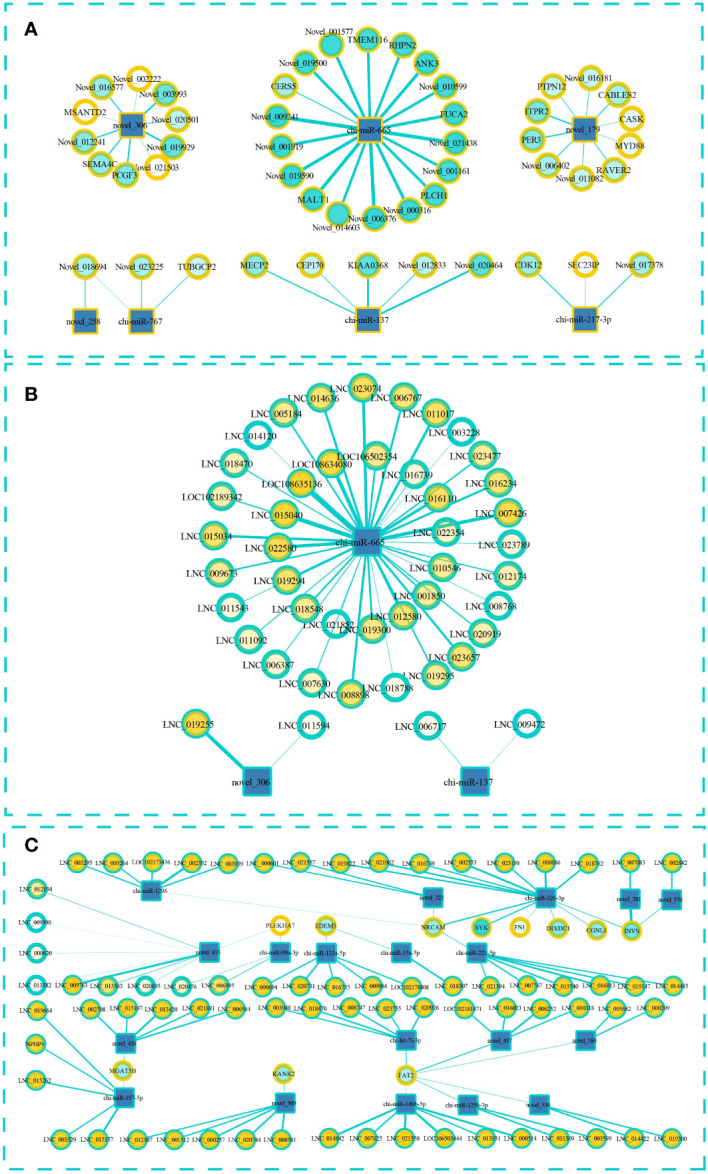
Interaction regulation network about miRNA–lncRNA–mRNA. **(A)** Targeted mRNA is predicted based on differentially expressed miRNA. **(B)** Targeted lncRNA is predicted based on differentially expressed miRNA. **(C)** The ceRNA interaction network of lncRNA–miRNA–mRNA in which miRNA and lncRNA were screened according to the differentially expressed mRNA. The square represents miRNA, the blue outer ring circle represents lncRNA, and the yellow outer ring circle represents mRNA. The thickness of the wire represents the absolute value of the predicted interaction correlation, and the color inside the circle indicates the p-value.

To predict the targeting relationship of the known and novel miRNAs with the mRNAs and lncRNAs, we used MiRanda and qTar software. We calculated Pearson correlation coefficients and used negative correlations to construct miRNA–lncRNA and miRNA–mRNA regulatory networks. LncRNAs can bind to miRNAs and can act as sponges to regulate the effect of miRNAs on their target miRNA. Therefore, we also constructed a ternary ceRNA interaction network of lncRNA–miRNA–mRNA (**Figure 5**; **Supplementary Material S7**). After screening, 49 pairs miRNA–mRNA pairs were predicted and the participating miRNAs were significantly differentially expressed between the low- and high-fecundity groups (**Figure 5A**). The novel_179–*ITPR2*, novel_179–*PER3*, novel_179–Novel_011082, and novel_179–*RAVER2* pairs were of particular interest because the target mRNAs were also significantly differentially expressed between the low- and high-fecundity groups *(ITPR2* q ≤0.01, *PER3* q ≤0.05, Novel_011082 q ≤0.05, and *RAVER2* q ≤0.05). Similarly, 45 miRNA–lncRNA were predicted and three of the participating lncRNAs were significantly differentially expressed between the low- and high-fecundity groups, namely, LNC_019255 (q ≤0 01), LNC_006767 (q ≤0 01), and LNC_019300 (q ≤0 01) (**Figure 5B**).

On the basis of the relationships between the miRNAs, lncRNAs, and mRNAs, we successfully constructed a ceRNA interaction network with 108 edges that contained 19 miRNAs, 11 mRNAs, and 73 lncRNAs (**Figure 5C**). The transcripts of 10 genes in the network were significantly differentially expressed between the high- and low-fecundity groups, namely, *PLEKHA7*-XM_018059600.1 (log2FC=−9.332, q ≤0.01), *FAT2*-XM_018050131.1 (log2FC=−8.679, q ≤0.01), *EDEM3*-XM_018060712.1 (log2FC=−2.254, q ≤0.01), *KANK2*-XM_018051102.1 (log2FC=−1.727, q ≤0.05), *MGAT5B*-XM_018063721.1 (log2FC=1.543, q ≤0.01), *FN1*-XM_018059847.1 (log2FC=3.909, q ≤0.01), *DIXDC1*-XM_018059788.1 (log2FC=−3.580, q ≤0.01), *SYK*-XM_005684185.3 (log2FC=4.380, q ≤0.05), *NRCAM*-XM_018047218.1 (log2FC=8.804, q ≤0.01), and *CGNL1*-XM_018054269.1 (log2FC=8.938, q ≤0.05). The Pearson correlation coefficient results indicated that it was highly possible that the binding of novel_281 and LNC_007083 affected the expression of *INVS* mRNA and that the binding of chi-miR-326-3p and lncRNA affected the expression of *SYK* and *NRCAM* mRNAs. However, the differential expression analysis of the transcripts of *INVS* showed that only one of the *INVS* variants (GenBank: XM_013966062.2) was significantly differentially expressed; the transcript of the other *INVS* variant (GenBank: XM_013966063.2) was not significantly differentially expressed (**Supplementary Material S7**).

### Validation of RNA sequencing using RT-qPCR

**Figure 6:**
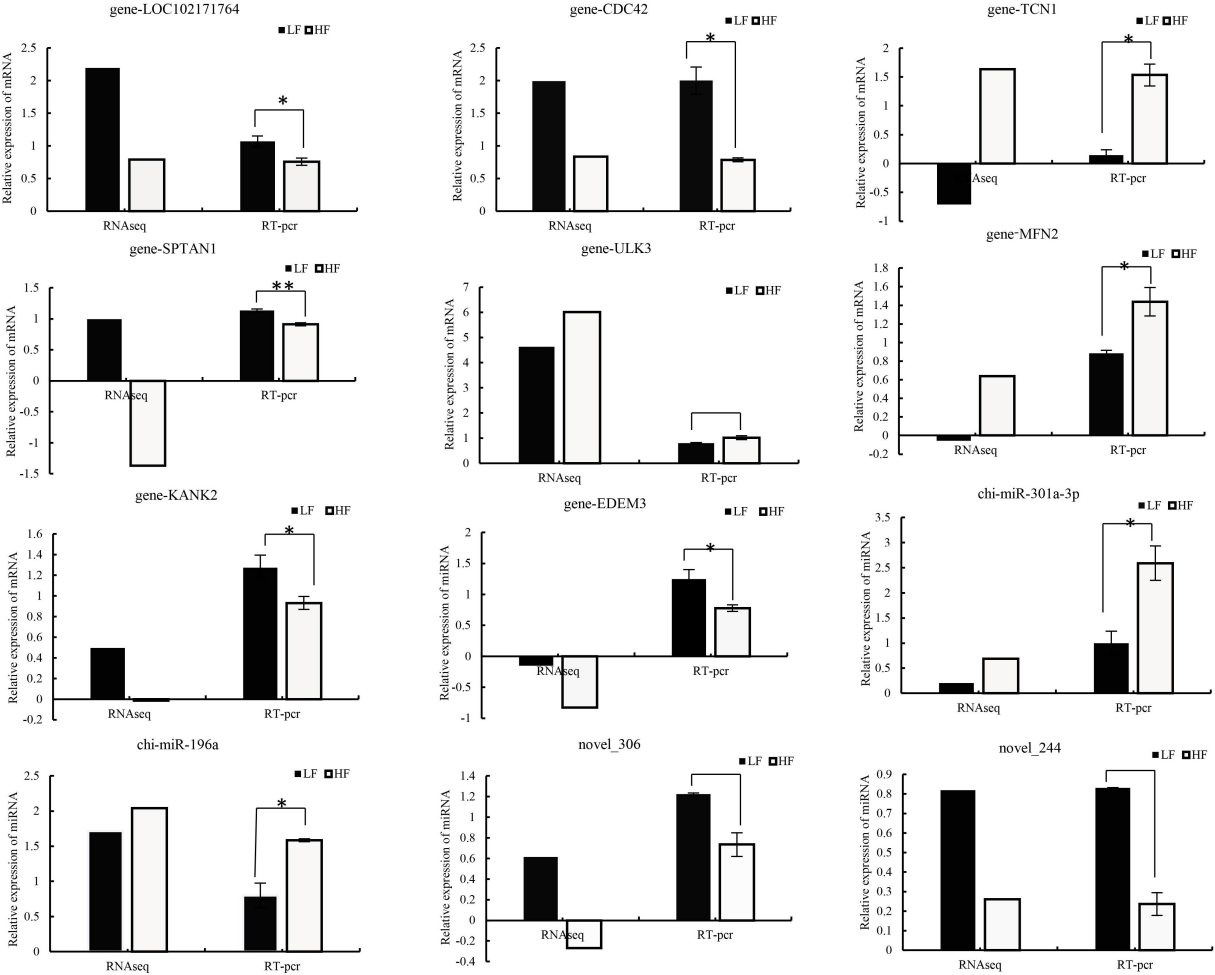
Comparison of real-time quantitative PCR (RT–qPCR) and RNA-seq results for validation of differentially expressed DEGs (n=8) and DEMs (n=4). Relative gene expression was calculated using the 2^−△△CP^ method, FPKM (mRNA), and TPM (miRNA).

To verify the accuracy and stability of sequencing results, RT-qPCR was used to compare the RNA-seq data. From the results, the quantitative results of mRNA and miRNA are consistent with the sequencing results (**Figure 6**), which certified the reliability of the data produced by sequencing. Relative gene expression was calculated using the 2^−△△CP^ method.

## Discussion

The Yunshang black goat is a new breed of mutton sheep that was bred in China using the black Nubi goat as the male parent and the Yunling black goat as the female parent after decades of crossbreeding (5, 20). Yunshang black goats have good meat production performance and delicious meat and are very popular in China, but high kidding number is the most wanted reproductive trait. Although kidding traits of Yunshang black goat have been subjected to strong positive selection during decades of cultivation (5), intravarietal significant differences in kidding number are still found. A statistical analysis of kidding numbers showed that the high-fecundity group had an average kidding number of 3.00 ± 0.38 and the low-fecundity group had an average kidding number of 1.32 ± 0.19 (p <0.05) (35). There was also a significant difference in the kidding number in this study but no significant difference in the number of follicles. Thus, pregnancy loss may be the key to affect fecundity. Many candidate genes have been shown to affect the litter size of sheep, including the main fecundity gene *FecB*, but when CRISPR-Cas technology was used to knock out one of the candidate genes BMPR-IB, it had no significant effect on the kidding number of goats (36). Until now, the major gene that affects the kidding number of goat has not been found. Many candidate genes related to kidding number have been discovered in goat, but the related molecular mechanism is not very clear; for example, some targeted genes (e.g., *AMHR2*, *FGFR1*, and *SMAD2*) under selection were reported in a previous study (37). Genomic regions linked to fecundity were indirectly identified by comparing two typical breeds and combining F-statistics (Fst) and heterozygosity (Hp) (38). Genomic selection sweep analysis and selection signatures analysis have been performed in single goat breeds with differing kidding numbers (39–42). In this study, we used Yunshang black goats with low and high kidding numbers to explore these differences and find candidate genes.

Successful pregnancy depends on a well-developed embryo and uterus with the right environment (43). Before the embryo and uterus can establish a connection, they undergo a preparation process. For example, the cytokine IFNT (IFNT a type I IFN), which is secreted by the trophoblast cells of the conceptus and has strong anti-viral, anti-proliferation, and immune-regulating effects, is required by the ruminant mother to recognize the pregnancy signal (10, 44). The mother also needs to secrete various hormones to regulate the uterine environment to cope with implantation of the conceptus; estrogen and progesterone are the most critical of these hormones (45). Successful attachment of the embryo to the uterine lining is needed before subsequent development can occur. Different from other ruminants, in goats, the attachment method is superficial implantation, and therefore, the embryo does not invade the endometrium (11). Opposition of the conceptus involves the trophectoderm becoming closely associated with the endometrial luminal epithelium followed by unstable adhesion. After day 14 of mating, the filamentous conceptus appears to be immobilized in the uterine lumen, and the trophectoderm maintains close contact with the endometrial luminal epithelium (46). Close association of the apical membranes of both these cell types takes place; however, the conceptus can still be recovered intact from the uterus by lavage. Opposition of the blastocyst is ensured by the interdigitation of cytoplasmic projections of the trophectoderm cells and uterine epithelial microvilli (9). In ruminants, the openings of uterine glands are also sites of apposition (47). The trophoblast develops fingerlike villi or papillae between the caruncles, which penetrate the mouths of the superficial ducts of the uterine glands at days 15–18 after mating (9, 48). During their short life (the villi/papillae disappear by day 20), these trophoblastic differentiations were hypothesized to anchor the per-attachment conceptus and absorb phototrophic secretions of the glands (9). Similar features have been described for the cow conceptus from day 15 of pregnancy, but, curiously, the goat conceptus lacks trophoblast papillae. In this study, we performed enrichment analysis on DEGs and differentially expressed target genes and found that adhesion junction, focal adhesion, and related pathways such as epidermal growth factor were significantly enriched. A recent study that used cervical mucus of different breeds of ewes with high and low fecundity for physicochemical analysis and RNA sequencing found significant differences in the terminal modification of mucin between the different fecundities and that the differentially expressed genes were related to adhesion (49).

Our sequencing results show that there were significant differences in the expression of hub mRNA between the miRNA–mRNA interaction network and the miRNA–lncRNA–mRNA ceRNA network. Among them, *PLEKHA7*, which encodes PLEKHA7 (pleckstrin homology domain-containing A7), is a zonula adhesion component closely connected with Nezha, which anchors the microtubules of epithelial cells to the zonula adhesion junction (50). Pulimeno et al. (51) showed that PLEKHA7 was located only in the apical part of epithelial cells in several tissues and that PLEKHA7 was involved in the recruitment of E-cadherin to enhance the potential of adhesion (52). The endometrium is made up of epithelial cells and, in goat, which mainly show surface attachment during embryo implantation, a cotyledon placenta is finally formed by adhesion to the endometrium. Therefore, adhesion ability has a key role for successful pregnancy. Whether our results can confirm that the difference between the high- and low-fecundity goats in our study is due to a difference in endometrial adhesion ability needs further analysis. Besides *PLEKHA7*, which is involved in adhesion, it has been reported that CircPlekha7, which is derived from *PLEKHA7*, also plays an important role in resisting pathological intrauterine adhesion (53). Fibronectin 1 (FN1), a large extracellular matrix adhesion glycoprotein composed of two similar subunits, has a variety of cell surface and extracellular ligand-binding domains and is involved in a many biological processes, including intrauterine adhesion (54). For example, *FN1* and its receptors have different functions in mammalian reproduction and embryogenesis (sperm–oocyte interaction, implantation, and placenta formation), and most FN1 receptors are integrins that transmit cellular signals in both directions (55–57). The mRNA that encodes the spleen tyrosine kinase (SYK) protein, which is part of the integrin cluster (58), was differentially expressed in our study. One of its functions is to stimulate the phosphorylation of Toll-like receptor 4 (TLR4) (59), which recognizes bacterial lipopolysaccharide and induces inflammation and immune response. SYK also induces pathogen recognition, cell adhesion, and platelet activation (60). Embryo implantation is known to be an immune response to the mother.

The surface attachment mode of goat embryos is also the attachment mode of other ruminant embryos and is very different from that of other mammals. The main difference lies in the fetal–maternal cell fusion process. There are three types of trophoblasts in the bovine placenta: mononucleate trophoblast cells (MTCs), binucleate cells (BNCs), and trinucleate cells (TNCs) (61). MTCs and BNCs have consistently been identified in the placental trophectoderm of many ruminants, whereas TNCs have been found only in bovine species, including cattle (62). Instead of TNCs, sheep and goats, which belonging to subfamily Caprinae (family Bovidae), develop multinucleated syncytial plaques (SyPs) (63). BNCs are formed by differentiation of MTCs by endoreduplication, whereas TNCs and multinucleated syncytial plaques are thought to be generated by cell-to-cell fusion between BNCs and maternal endometrial cells (64). Nakaya et al. (62) found that a retrovirus located in intron 18 of the bovine FAT2 gene played a significant role in bovine TNC formation. Coincidentally, we detected a difference in the expression of FAT2 between the high- and low-fecundity groups. In addition, the differentially expressed *ITPR2* gene in our study encodes inositol 1,4,5-trisphosphate receptor type 2 (IP3R2), which is a member of the family of intracellular Ca^2+^ release channels and is located on the endoplasmic reticulum membrane (65). Studies using a gene knockout mouse model have shown that IP3R plays important roles in regulating many physiological processes, including embryonic survival (66), extraembryonic vascular development (67), T-cell development (68), and B-cell function (69). Allantoic-placental defects were detected in all IP_3_R_2_ and IP_3_R_3_ knockout embryos, and the fetal cardiovascular development was abnormal under the influence of placental defects (65). It has also been suggested that the Ca^2+^ signaling pathway may be involved in regulating the establishment of fetal–maternal connections (70).

## Conclusions

We identified 1,674 differentially expressed mRNAs (914 upregulated and 760 downregulated), 288 differentially expressed lncRNAs (149 upregulated and 139 downregulated), and 17 differentially expressed miRNAs (4 upregulated and 13 downregulated) in low-fecundity vs. high-fecundity comparisons. By constructing interaction networks, we discovered 49 pairs in the miRNA–mRNA network and 45 pairs in the miRNA–lncRNA network. We successfully constructed a ceRNA interaction network with 108 edges that was composed of 19 miRNAs, 11 mRNAs, and 73 lncRNAs. Five candidate genes (*PLEKHA7*, *FAT2*, *FN1*, *SYK*, and *ITPR2*) related to cell adhesion or calcium membrane channel protein were identified. Our study provides the overall expression profiles of mRNAs, lncRNA, and miRNA in goat uterus during the proliferative period and offers new insights into the mechanisms associated with the high fecundity of goats, which may be helpful to guide goat to reduce pregnancy loss.

## Data availability statement

The datasets presented in this study can be found in online repositories. The names of the repository/repositories and accession number(s) can be found in the article/**Supplementary Material**.

## Ethics statement

This manuscript follows the ARRIVE animal experiment report guidelines. The animal study was reviewed and approved by the Animal Ethics Committee of the Institute of Animal Sciences of the Chinese Academy of Agricultural Sciences (No. IAS2019-63). Written informed consent was obtained from the owners for the participation of their animals in this study.

## Author contributions

Conceptualization: MC and MF. Methodology: XD, YL, MC, and MF. Validation: XD, YL, and XH. Formal analysis: XD and MC. Resources: XD, YL, LT, and MC. Investigation: XD, YL, LT, XH, MF, and MC. Writing—original draft preparation: XD and MC. Data curation: XD, YL, LT, MF, and MC. Supervision: MC. Project administration, MC. Funding acquisition: MC. All authors have read and agreed to the published version of the manuscript.

## References

[1] Wang XY Guo XF He XY Liu QY Di R Hu WP. Effects of FecB mutation on estrus, ovulation, and endocrine characteristics in small tail han sheep. Front Vet Sci (2021) 8. doi: 10.3389/fvets.2021.709737 PMC8646036 34881317

[2] Yang QY Liu J Wang Y Zhao W Wang WJ Cui J. A proteomic atlas of ligand-receptor interactions at the ovine maternal-fetal interface reveals the role of histone lactylation in uterine remodeling. J Biol Chem (2022) 298(1):101456. doi: 10.1016/j.jbc.2021.101456 34861240 PMC8733267

[3] Wang HB Dey SK. Roadmap to embryo implantation: clues from mouse models. Nat Rev Genet (2006) 7(3):185–99. doi: 10.1038/nrg1808 16485018

[4] Norwitz ER Schust DJ Fisher SJ. Mechanisms of disease - implantation and the survival of early pregnancy. New Engl J Med (2001) 345(19):1400–8. doi: 10.1056/NEJMra000763 11794174

[5] Tao L He XY Jiang YT Lan R Li M Li ZM. Combined approaches to reveal genes associated with litter size in yunshang black goats. Anim Genet (2020) 51(6):924–34. doi: 10.1111/age.12999 32986880

[6] Spencer TE Johnson GA Burghardt RC Bazer FW. Progesterone and placental hormone actions on the uterus: insights from domestic animals. Biol Reprod (2004) 71(1):2–10. doi: 10.1095/biolreprod.103.024133 14973264

[7] Homer H Rice GE Salomon C. Review: embryo- and endometrium-derived exosomes and their potential role in assisted reproductive treatments-liquid biopsies for endometrial receptivity. Placenta (2017) 54:89–94. doi: 10.1016/j.placenta.2016.12.011 28043656

[8] Cha J Dey SK. Cadence of procreation: orchestrating embryo-uterine interactions. Semin Cell Dev Biol (2014) 34:56–64. doi: 10.1016/j.semcdb.2014.05.005 24862857 PMC4163119

[9] Guillomot M Flechon JE Wintenberger-Torres S. Conceptus attachment in the ewe: an ultrastructural study. Placenta (1981) 2(2):169–82. doi: 10.1016/S0143-4004(81)80021-5 7232339

[10] Spencer TE Hansen TR. Implantation and establishment of pregnancy in ruminants. Adv Anat Embryol Cell Biol (2015) 216:105–35. doi: 10.1007/978-3-319-15856-3_7 26450497

[11] Wango EO Wooding FB Heap RB. The role of trophoblastic binucleate cells in implantation in the goat: a morphological study. J Anat (1990) 171:241–57. PMC1257145 1707046

[12] Gray CA Burghardt RC Johnson GA Bazer FW Spencer TE. Evidence that absence of endometrial gland secretions in uterine gland knockout ewes compromises conceptus survival and elongation. Reproduction (2002) 124(2):289–300. doi: 10.1530/rep.0.1240289 12141942

[13] Munro SK Farquhar CM Mitchell MD Ponnampalam AP. Epigenetic regulation of endometrium during the menstrual cycle. Mol Hum Reprod (2010) 16(5):297–310. doi: 10.1093/molehr/gaq010 20139117

[14] Hong XM Luense LJ McGinnis LK Nothnick WB Christenson LK. Dicer1 is essential for female fertility and normal development of the female reproductive system. Endocrinology (2008) 149(12):6207–12. doi: 10.1210/en.2008-0294 PMC2613048 18703631

[15] Toloubeydokhti T Pan Q Luo X Bukulmez O Chegini N. The expression and ovarian steroid regulation of endometrial micro-RNAs. Reprod Sci (2014) 21(10):1326–6. doi: 10.1177/1933719114547496 PMC2729333 19088369

[16] Zhang L Liu XR Liu JZ Zhou ZQ Song YX Cao BY. miR-182 aids in receptive endometrium development in dairy goats by down-regulating PTN expression. PLoS One (2017) 12(7):e0179783. doi: 10.1371/journal.pone.0179783 28678802 PMC5497977

[17] Zhang L Liu XR Liu JZ Song YX Zhou ZQ Cao BY. miR-182 selectively targets HOXA10 in goat endometrial epithelium cells *in vitro* . Reprod Domest Anim (2017) 52(6):1081–92. doi: 10.1111/rda.13031 28758253

[18] Xu Z Hu Q Zang XP Zhou C Liu DW Liu GB. Analysis of transcripts of uncertain coding potential using RNA sequencing during the preattachment phase in goat endometrium. DNA Cell Biol (2021) 40(7):998–1008. doi: 10.1089/dna.2020.6463 34115954

[19] Hong LJ Hu Q Zang XP Xie YS Zhou C Zou X. Analysis and screening of reproductive long non-coding RNAs through genome-wide analyses of goat endometrium during the pre-attachment phase. Front Genet (2020) 11. doi: 10.3389/fgene.2020.568017 PMC7649795 33193661

[20] Liang C Han MC Zhou ZY Liu YF He XY Jiang YT. Hypothalamic transcriptome analysis reveals the crucial MicroRNAs and mRNAs affecting litter size in goats. Front Vet Sci (2021) 8. doi: 10.3389/fvets.2021.747100 PMC8591166 34790713

[21] Pertea M Pertea GM Antonescu CM Chang TC Mendell JT Salzberg SL. StringTie enables improved reconstruction of a transcriptome from RNA-seq reads. Nat Biotechnol (2015) 33(3):290. doi: 10.1038/nbt.3122 25690850 PMC4643835

[22] Pertea M Kim D Pertea GM Leek JT Salzberg SL. Transcript-level expression analysis of RNA-seq experiments with HISAT, StringTie and ballgown. Nat Protoc (2016) 11(9):1650–67. doi: 10.1038/nprot.2016.095 PMC5032908 27560171

[23] Trapnell C Williams BA Pertea G Mortazavi A Kwan G van Baren MJ. Transcript assembly and quantification by RNA-seq reveals unannotated transcripts and isoform switching during cell differentiation. Nat Biotechnol (2010) 28(5):511–U174. doi: 10.1038/nbt.1621 20436464 PMC3146043

[24] Sun L Luo HT Bu DC Zhao GG Yu KT Zhang CH. Utilizing sequence intrinsic composition to classify protein-coding and long non-coding transcripts. Nucleic Acids Res (2013) 41(17):e166. doi: 10.1093/nar/gkt646 23892401 PMC3783192

[25] Kong L Zhang Y Ye ZQ Liu XQ Zhao SQ Wei L. CPC: assess the protein-coding potential of transcripts using sequence features and support vector machine. Nucleic Acids Res (2007) 35:W345–9. doi: 10.1093/nar/gkm391 PMC1933232 17631615

[26] Li AM Zhang JY Zhou ZY. PLEK: a tool for predicting long non-coding RNAs and messenger RNAs based on an improved k-mer scheme. BMC Bioinf (2014) 15:311. doi: 10.1186/1471-2105-15-S8-S1 PMC4177586 25239089

[27] Griffiths-Jones S. miRBase: the microRNA sequence database. Methods Mol Biol (2006) 342:129–38. doi: 10.1385/1-59745-123-1:129 16957372

[28] Burge SW Daub J Eberhardt R Tate J Barquist L Nawrocki EP. Rfam 11.0: 10 years of RNA families. Nucleic Acids Res (2013) 41(D1):D226–32. doi: 10.1093/nar/gks1005 PMC3531072 23125362

[29] Huda A Jordan IK. Analysis of transposable element sequences using CENSOR and RepeatMasker. Bioinf DNA Sequence Anal (2009) 537:323–36. doi: 10.1007/978-1-59745-251-9_16 19378152

[30] Quinlan AR Hall IM. BEDTools: a flexible suite of utilities for comparing genomic features. Bioinformatics (2010) 26(6):841–2. doi: 10.1093/bioinformatics/btq033 PMC2832824 20110278

[31] Friedlander MR Mackowiak SD Li N Chen W Rajewsky N. miRDeep2 accurately identifies known and hundreds of novel microRNA genes in seven animal clades. Nucleic Acids Res (2012) 40(1):37–52. doi: 10.1093/nar/gkr688 21911355 PMC3245920

[32] Love MI Huber W Anders S. Moderated estimation of fold change and dispersion for RNA-seq data with DESeq2. Genome Biol (2014) 15(12):550. doi: 10.1186/s13059-014-0550-8 25516281 PMC4302049

[33] Xie C Mao XZ Huang JJ Ding Y Wu JM Dong S. KOBAS 2.0: a web server for annotation and identification of enriched pathways and diseases. Nucleic Acids Res (2011) 39:W316–22. doi: 10.1093/nar/gkr483 PMC3125809 21715386

[34] Jung JE Lee JY Park HR Kang JW Kim YH Lee JH. MicroRNA-133 targets phosphodiesterase 1C in drosophila and human oral cancer cells to regulate epithelial-mesenchymal transition. J Cancer (2021) 12(17):5296–309. doi: 10.7150/jca.56138 PMC8317528 34335946

[35] Liu YF Chen YL Zhou ZY He XY Tao L Jiang YT. Chi-miR-324-3p regulates goat granulosa cell proliferation by targeting DENND1A. Front Vet Sci (2021) 8. doi: 10.3389/fvets.2021.732440 PMC8636700 34869714

[36] Kumar S Punetha M Jose B Bharati J Khanna S Sonwane A. Modulation of granulosa cell function via CRISPR-cas fuelled editing of BMPR-IB gene in goats (Capra hircus). Sci Rep-Uk (2020) 10(1):20446. doi: 10.1038/s41598-020-77596-9 PMC7686318 33235250

[37] Lai FN Zhai HL Cheng M Ma JY Cheng SF Ge W. Whole-genome scanning for the litter size trait associated genes and SNPs under selection in dairy goat (Capra hircus). Sci Rep-Uk (2016) 6:38096. doi: 10.1038/srep38096 PMC5131482 27905513

[38] Guan DL Luo NJ Tan XS Zhao ZQ Huang YF Na RS. Scanning of selection signature provides a glimpse into important economic traits in goats (Capra hircus). Sci Rep-Uk (2016) 6:36372. doi: 10.1038/srep36372 PMC5087083 27796358

[39] GX E Duan XH Zhang JH Huang YF Zhao YJ Na RS. Genome-wide selection signatures analysis of litter size in dazu black goats using single-nucleotide polymorphism. 3 Biotech (2019) 9(9):336. doi: 10.1007/s13205-019-1869-3 PMC6702510 31475088

[40] Guang-Xin E Zhu YB Basang WD Na RS Han YG Zeng Y. Comparative and selection sweep analysis of CNV was associated to litter size in dazu black goats. Anim Biotechnol (2021) 32(6):792–7. doi: 10.1080/10495398.2020.1753756 32293982

[41] Guang-Xin E Zhao YJ Huang YF. Selection signatures of litter size in dazu black goats based on a whole genome sequencing mixed pools strategy. Mol Biol Rep (2019) 46(5):5517–23. doi: 10.1007/s11033-019-04904-6 31175513

[42] Wang JJ Zhang T Chen QM Zhang RQ Li L Cheng SF. Genomic signatures of selection associated with litter size trait in jining Gray goat. Front Genet (2020) 11. doi: 10.3389/fgene.2020.00286 PMC7113370 32273886

[43] Paria BC Huet-Hudson YM Dey SK. Blastocyst's state of activity determines the "window" of implantation in the receptive mouse uterus. Proc Natl Acad Sci U.S.A. (1993) 90(21):10159–62. doi: 10.1073/pnas.90.21.10159 PMC47733 8234270

[44] Bazer FW Wu G Spencer TE Johnson GA Burghardt RC Bayless K. Novel pathways for implantation and establishment and maintenance of pregnancy in mammals. Mol Hum Reprod (2010) 16(3):135–52. doi: 10.1093/molehr/gap095 PMC2816171 19880575

[45] Zhang S Lin HY Kong SB Wang SM Wang HM Wang HB. Physiological and molecular determinants of embryo implantation. Mol Aspects Med (2013) 34(5):939–80. doi: 10.1016/j.mam.2012.12.011 PMC4278353 23290997

[46] King GJ Atkinson BA Robertson HA. Implantation and early placentation in domestic ungulates. J Reprod Fertil Suppl (1982) 31:17–30. 6762430

[47] Guillomot M Guay P. Ultrastructural features of the cell surfaces of uterine and trophoblastic epithelia during embryo attachment in the cow. Anat Rec (1982) 204(4):315–22. doi: 10.1002/ar.1092040404 7181136

[48] Wooding FB. The role of the binucleate cell in ruminant placental structure. J Reprod Fertil Suppl (1982) 31:31–9. 6762432

[49] Abril-Parreno L Morgan J Krogenaes A Druart X Cormican P Gallagher ME. Biochemical and molecular characterisation of sialylated cervical mucins in sheep. Biol Reprod (2022) 107:419–31. doi: 10.1093/biolre/ioac077 PMC9382375 35470857

[50] Meng W Mushika Y Ichii T Takeichi M. Anchorage of microtubule minus ends to adherens junctions regulates epithelial cell-cell contacts. Cell (2008) 135(5):948–59. doi: 10.1016/j.cell.2008.09.040 19041755

[51] Pulimeno P Bauer C Stutz J Citi S. PLEKHA7 is an adherens junction protein with a tissue distribution and subcellular localization distinct from ZO-1 and e-cadherin. PLoS One (2010) 5(8):e12207. doi: 10.1371/journal.pone.0012207 20808826 PMC2924883

[52] Citi S Pulimeno P Paschoud S. Cingulin, paracingulin, and PLEKHA7: signaling and cytoskeletal adaptors at the apical junctional complex. Ann Ny Acad Sci (2012) 1257:125–32. doi: 10.1111/j.1749-6632.2012.06506.x 22671598

[53] Xie W He M Liu YH Huang XW Song DM Xiao Y. CircPlekha7 plays an anti-fibrotic role in intrauterine adhesions by modulating endometrial stromal cell proliferation and apoptosis. J Reprod Dev (2020) 66(6):493–504. doi: 10.1262/jrd.2019-165 32801258 PMC7768166

[54] Goossens K Van Soom A Van Zeveren A Favoreel H Peelman LJ. Quantification of fibronectin 1 (FN1) splice variants, including two novel ones, and analysis of integrins as candidate FN1 receptors in bovine preimplantation embryos. BMC Dev Biol (2009) 9:1–11. doi: 10.1186/1471-213X-9-1 PMC2648952 19126199

[55] Wang J Armant DR. Integrin-mediated adhesion and signaling during blastocyst implantation. Cells Tissues Organs (2002) 172(3):190–201. doi: 10.1159/000066970 12476048

[56] Wang J Mayernik L Armant DR. Trophoblast adhesion of the peri-implantation mouse blastocyst is regulated by integrin signaling that targets phospholipase c. Dev Biol (2007) 302(1):143–53. doi: 10.1016/j.ydbio.2006.09.015 PMC1894903 17027741

[57] George EL Georges-Labouesse EN Patel-King RS Rayburn H Hynes RO. Defects in mesoderm, neural tube and vascular development in mouse embryos lacking fibronectin. Development (1993) 119(4):1079–91. doi: 10.1242/dev.119.4.1079 8306876

[58] Luigi-Sierra MG Fernandez A Martinez A Guan DL Delgado JV Alvarez JF. Genomic patterns of homozygosity and inbreeding depression in murciano-granadina goats. J Anim Sci Biotechno (2022) 13(1):35. doi: 10.1186/s40104-022-00684-5 PMC8908635 35264251

[59] Miller YI Choi SH Wiesner P Bae YS. The SYK side of TLR4: signalling mechanisms in response to LPS and minimally oxidized LDL. Brit J Pharmacol (2012) 167(5):990–9. doi: 10.1111/j.1476-5381.2012.02097.x PMC3492981 22776094

[60] Mocsai A Ruland J Tybulewicz VLJ. The SYK tyrosine kinase: a crucial player in diverse biological functions. Nat Rev Immunol (2010) 10(6):387–402. doi: 10.1038/nri2765 20467426 PMC4782221

[61] Klisch K Pfarrer C Schuler G Hoffmann B Leiser R. Tripolar acytokinetic mitosis and formation of feto-maternal syncytia in the bovine placentome: different modes of the generation of multinuclear cells. Anat Embryol (1999) 200(2):229–37. doi: 10.1007/s004290050275 10424879

[62] Nakaya Y Koshi K Nakagawa S Hashizume K Miyazawa T. Fematrin-1 is involved in fetomaternal cell-to-Cell fusion in bovinae placenta and has contributed to diversity of ruminant placentation. J Virol (2013) 87(19):10563–72. doi: 10.1128/JVI.01398-13 PMC3807419 23864631

[63] Black SG Arnaud F Palmarini M Spencer TE. Endogenous retroviruses in trophoblast differentiation and placental development. Am J Reprod Immunol (2010) 64(4):255–64. doi: 10.1111/j.1600-0897.2010.00860.x PMC4198168 20528833

[64] Klisch K Hecht W Pfarrer C Schuler G Hoffmann B Leiser R. DNA Content and ploidy level of bovine placentomal trophoblast giant cells. Placenta (1999) 20(5-6):451–8. doi: 10.1053/plac.1999.0402 10419810

[65] Yang FL Huang L Tso A Wang H Cui L Lin LZ. Inositol 1,4,5-trisphosphate receptors are essential for fetal-maternal connection and embryo viability. PLoS Genet (2020) 16(4):e1008739. doi: 10.1371/journal.pgen.1008739 32320395 PMC7176088

[66] Nakazawa M Uchida K Aramaki M Kodo K Yamagishi C Takahashi T. Inositol 1,4,5-trisphosphate receptors are essential for the development of the second heart field. J Mol Cell Cardiol (2011) 51(1):58–66. doi: 10.1016/j.yjmcc.2011.02.014 21382375

[67] Uchida K Nakazawa M Yamagishi C Mikoshiba K Yamagishi H. Type 1 and 3 inositol trisphosphate receptors are required for extra-embryonic vascular development. Dev Biol (2016) 418(1):89–97. doi: 10.1016/j.ydbio.2016.08.007 27514653

[68] Ouyang KF Gomez-Amaro RL Stachura DL Tang HY Peng XH Fang X. Loss of IP3R-dependent Ca2+ signalling in thymocytes leads to aberrant development and acute lymphoblastic leukemia. Nat Commun (2014) 5:4814. doi: 10.1038/ncomms5814 25215520 PMC5537137

[69] Tang HY Wang H Lin QS Fan FF Zhang F Peng XH. Loss of IP3 receptor-mediated Ca2+ release in mouse b cells results in abnormal b cell development and function. J Immunol (2017) 199(2):570–80. doi: 10.4049/jimmunol.1700109 28615414

[70] Nakamura Y Hamada Y Fujiwara T Enomoto H Hiroe T Tanaka S. Phospholipase c-delta1 and -delta3 are essential in the trophoblast for placental development. Mol Cell Biol (2005) 25(24):10979–88. doi: 10.1128/MCB.25.24.10979-10988.2005 PMC1316982 16314520

